# Clinical Neuropathology Practice News 4-2012: levels of evidence for brain tumor biomarkers 

**DOI:** 10.5414/NP300511

**Published:** 2012-06-19

**Authors:** Anna Sophie Berghoff, Harald Stefanits, Harald Heinzl, Matthias Preusser

**Affiliations:** 1 Institute of Neurology and Comprehensive Cancer Center CNS Unit,; 2 Center for Medical Statistics, Informatics, and Intelligent Systems,; 3 Department of Medicine I and Comprehensive Cancer Center CNS Unit, Medical University of Vienna, Vienna, Austria

**Keywords:** brain tumors, level of evidence, biomarker, glioma

## Abstract

The National Comprehensive Cancer Network (NCCN) recently published a task force report on the evaluation of the clinical utility of tumor biomarkers in oncology. In this report, common terminology and the use of levels of evidence scores to aid the evaluation of biomarker tests in oncology were proposed. Furthermore, the task force applied a level of evidence system to selected biomarkers of several cancer types. According to this system, the highest level of evidence, IA, is granted to a biomarker only if it has been evaluated in at least one adequately powered and specifically designed prospective controlled trial. For gliomas, only 1p/19q testing in oligodendroglial tumors was classified as IA by the NCCN task force. For all of the following biomarkers the present evidence level for clinical utility was regarded as lower than that of 1p/19q status: MGMT gene promoter methylation testing (glioblastoma), IDH mutation testing (diffusely growing gliomas), BRAF fusion testing (pilocytic astrocytoma) and CIMP testing (diffusely growing gliomas). The task force acknowledged that the exact application of levels of evidence needs further refinement. To our mind, the implementation of a brain tumor expert panel seems vital to evaluate the evidence levels of neurooncological biomarkers according to generally accepted criteria on a regular basis. Systematic identification of current research needs and widely accepted up-to-date recommendations for efficient biomarker application in everyday practice could be gained.

## Background 

In clinical medicine, biomarkers are defined as objectively measurable factors that provide information about a disease state, thus providing the possibility for diagnosis, prediction of patient outcome, choice of therapy or follow-up. A myriad of potential biomarkers have been proposed for gliomas, but almost none have translated into actual clinical use so far [1]. For some biomarkers, there is controversy about their clinical value. For example, O6-mehthylguanine-methyltransferase (MGMT) gene promoter methylation status is considered as predictive factor by some, while others regard it rather as prognostic parameter [2, 3]. Some criticize that, either way, the lack of consensus on the test to use and the undetermined or poor analytical performance (repeatability and reproducibility) of the various methods for MGMT testing significantly limits the clinical utility of this marker [4, 5, 6, 7]. 

The National Comprehensive Cancer Network (NCCN) recently published a task force report on the evaluation of the clinical utility of tumor biomarkers in oncology [8]. In this report, common terminology and the use of levels of evidence scores to aid the evaluation of biomarker tests in oncology were proposed. The group applied a level of evidence system to selected biomarkers of several tumor types including gliomas. This approach is of interest, as objective evaluation of biomarkers according to predefined criteria through an expert panel may facilitate research on and informed clinical use of biomarkers in neurooncology. 

## Terminology 

Biomarkers have different properties. This complexity necessitates the implementation of a commonly agreed upon terminology [8]. Table 1 contains selected terms with high relevance for neurooncology. From a neuropathology perspective it is important to note that for almost none of the potential glioma biomarker tests proposed in the literature stringent analysis of their analytical validity has been performed. There is a strong need for well controlled studies on the performance of the various biomarker assays including inter-laboratory round robin studies (ring trials), because the analytical validity of a test is a prerequisite for its clinical utility. 

## Level of evidence system 

Several level of evidence systems for assessing tumor biomarkers have been suggested in previous publications. The NCCN Task Force Group created their levels of evidence system from a combination of the revised Tumor Marker Utility Grading System (TMGUS) and the levels of evidence grade for using archived tissue [8]. The highest level of evidence IA is considered only for a biomarker evaluated in at least one adequately powered prospective controlled trial. 

## Application to glioma biomarkers 

According to the NCCN Task Force Report, only 1p/19q testing in oligodendroglial tumors meets the criteria for IA level of evidence among potential glioma biomarkers, because randomized controlled trials support clinical validity and clinical utility of the test, and a practical consensus guideline on performing 1p/19q FISH analysis provides analytic validation [8, 9, 10]. However, in our opinion prospective studies specifically designed to assess the clinical performance of 1p/19q status and systematic studies on the analytical performance of 1p/19q testing are formally missing, although prospective 1p/19q testing was amended during the conduct of the therapeutic trial EORTC 26951 in order to assess the relation of 1p/19q with progression-free and overall survival [9]. For all of the following glioma biomarkers the evidence level was regarded as lower than of 1p/19q status (IIB) by the task force based on currently available data: isocitrate dehydrogenase (IDH) mutation testing (diffusely growing gliomas) [11, 12], MGMT gene promoter methylation testing (glioblastoma) [13], v-RAF murine sarcoma viral oncogene homolog B1 (BRAF) fusion testing (pilocytic astrocytoma) [14] and CpG island methylator phenotype (CIMP) testing (diffusely growing gliomas) [15]. The group acknowledged that the exact application of levels of evidence needs further refinement. In our opinion, more strict assessment of the analytical validity of biomarker tests is of central importance in brain tumors. Furthermore, inclusion of additional commonly used and novel emerging markers such as Ki67 tumor cell proliferation index (gliomas) [16, 17, 18], INI1 protein loss (atypical teratoid/rhabdoid tumor) [19, 20] or BRAF V600E mutation status (melanoma brain metastases) [21, 22, 23] seems desirable. 

## Conclusions 

The adoption of biomarkers into everyday practice is challenging. For most potential glioma biomarkers, differences in the study design concerning the evaluation of analytic and clinical validity as well as the clinical utility make it difficult to draw unequivocal conclusions. As a result, only a small proportion of biomarkers have been adopted into everyday practice, whereas on enormous amount of biomarkers is stuck in the research pipeline. The NCCN Task Force Report applies for the first time levels of evidence to evaluate biomarkers in glioma. 

To our mind, the implementation of a brain tumor expert panel seems vital to evaluate the evidence levels of neurooncological biomarkers according to generally accepted criteria on a regular basis. Systematic identification of current research needs and widely accepted up-to-date recommendations for efficient biomarker application in everyday practice could be gained. 

**Table 1.** Terminology of biomarker definitions.

Terminus	Characterization	Example/comment	References
Diagnostic marker	Aids the sub-classification of a particular disease state	INI1 for AT/RT	[19, 20]
Prognostic marker	Association with some clinical outcome, such as overall survival or recurrence-free survival, independent of the treatment rendered	IDH mutations in glioma	[11, 12]
Predictive marker	Predicts the activity of a specific class or type of therapy and helps to make specific treatment decisions	BRAF V600E mutation in melanoma brain metastases	[22, 23]
Analytical performance	Accuracy, reliability, repeatability, and reproducibility of an assay	Analytical performance has not been sufficiently investigated for most biomarkers in neuroonocology	[1]
Clinical performance	Association of test result with clinical outcome	MGMT promoter methylation has shown correlation with patient survival times in several studies	[2, 3, 13]
Clinical utility	Ability to improve clinical decision-making and patient outcomes	MGMT promoter methylation is not used in the clinical setting in many centers, because the assay has shown insufficient analytical performance	[4, 6]
